# The Acetylcholine Esterase Inhibitor Donepezil Increases Dopamine Levels in the Nucleus Accumbens and Blocks the Alcohol Deprivation Effect in Rats

**DOI:** 10.1111/adb.70150

**Published:** 2026-03-29

**Authors:** Anna Loftén, Klara Danielsson, Louise Adermark, Bo Söderpalm, Mia Ericson

**Affiliations:** ^1^ Addiction Biology Unit, Department of Psychiatry and Neurochemistry, Institute of Neuroscience and Physiology, the Sahlgrenska Academy University of Gothenburg Gothenburg Sweden; ^2^ Department of Addiction Disorders Sahlgrenska University Hospital, Region Västra Götaland Gothenburg Sweden; ^3^ Department of Pharmacology, Institute of Neuroscience and Physiology, the Sahlgrenska Academy University of Gothenburg Gothenburg Sweden

**Keywords:** acetylcholine, alcohol use disorder, donepezil, dopamine, nucleus accumbens, rat

## Abstract

Alcohol use disorder is associated with substantial morbidity and mortality arising from both medical consequences and accidents. Currently available pharmacotherapies are limited, and novel treatment options are needed. One putative target for pharmacotherapy is the mesolimbic dopamine system and its major target, the nucleus accumbens. Mesolimbic dopamine signaling is regulated by multiple neurotransmitters, among which acetylcholine is a key modulator. This study investigated whether the acetylcholine esterase inhibitor donepezil affects basal and/or alcohol‐induced increases in extracellular dopamine levels in the nucleus accumbens, and whether it influences alcohol consumption in male Wistar rats. Extracellular dopamine levels were measured using in vivo microdialysis, while alcohol intake was assessed using an intermittent two‐bottle choice paradigm followed by alcohol deprivation. Systemic administration of donepezil increased extracellular dopamine levels in the nucleus accumbens, an effect blocked by local administration of the muscarinic antagonist scopolamine but not by the nicotinic antagonist mecamylamine, indicating a primarily muscarinic mechanism. Moreover, following donepezil pretreatment, alcohol administration did not produce any further increase in dopamine. Donepezil did not alter voluntary alcohol consumption under intermittent access conditions, but it fully abolished the alcohol deprivation effect. These findings suggest that donepezil modulates accumbal dopamine transmission via muscarinic receptors and may influence neurobiological mechanisms underlying relapse‐like drinking.

## Graphical abstract

This study examined the effects of donepezil on mesolimbic dopamine signalling and alcohol‐related behaviours in male rats. Donepezil increases nucleus accumbens dopamine via muscarinic mechanisms and prevented further dopamine elevation after alcohol administration. Although it did not reduce regular alcohol intake, it abolished relapse‐like drinking.

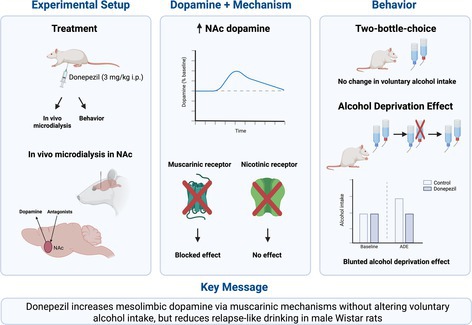

## 1 Introduction

Alcohol use disorder (AUD) is a prevalent and debilitating disorder that contributes substantially to global morbidity and mortality, accounting for approximately 5.6% of all global deaths annually [1, 2]. Despite its high prevalence and burden, pharmacological treatments for AUD remain limited, highlighting the need to develop novel pharmacotherapeutic options [3].

The mesolimbic dopamine (DA) system, a central component in the reward circuitry, consists of DA neurons originating from the ventral tegmental area (VTA), projecting to multiple brain regions, including the nucleus accumbens (NAc). Activation of this pathway increases extracellular DA in the NAc, a process closely associated with the reinforcing properties of addictive substances, including alcohol [4, 5]. Mesolimbic DA signaling is regulated by multiple neurotransmitters, both at the cell body level in the VTA and via presynaptic mechanisms in the NAc. Among these modulators, acetylcholine (ACh) plays a crucial role in both the VTA and NAc [6, 7, 8].

Cholinergic input to VTA arises from the pedunculopontine nucleus (PPN) and the laterodorsal tegmental nucleus (LDTg) [9]. Increased activity of these cholinergic pathways facilitates burst firing of midbrain DA neurons [10], whereas their inhibition reduces DA neuron activity [11]. Both muscarinic (mAChRs) and nicotinic (nAChRs) acetylcholine receptors contribute to this regulation [12]. At the terminal level, ACh modulates DA signaling in the NAc, where the cholinergic interneurons (CINs) provide the primary source of ACh, with a minor contribution from PPN and LDTg projections [13]. Selective activation of CINs induces local DA release both in vitro [14, 15, 16] and in vivo [17], independent of VTA DA neuron firing. This process is largely mediated through nAChRs [14, 15, 16].

Alcohol activates the mesolimbic DA system and increases extracellular DA in the NAc following both systemic and local administration [5, 18, 19]. This effect can be attenuated by blocking nAChRs in the VTA [7, 20, 21] and by combined inhibition of nAChRs and mAChRs in the NAc [6]. Beyond its neurochemical influence, cholinergic signaling also affects alcohol‐related behaviours. Targeting mAChRs using allosteric modulators or antagonists has been shown to reduce alcohol intake in rodents in various drinking paradigms [22, 23] and to block alcohol‐induced conditioned place preference [24]. Similarly, the nAChR modulator varenicline reduces alcohol intake in both rodents [25, 26] and humans [27, 28], and the acetylcholine esterase inhibitor (AChEI) galantamine reduces alcohol intake in individuals with AUD [29]. Collectively, these findings support the potential of cholinergic modulation as a therapeutic approach for AUD.

The aim of this study was to test the hypothesis that the selective and centrally acting AChEI, donepezil, influences NAc DA during alcohol exposure and affects alcohol intake and/or relapse‐like drinking. To test this hypothesis, we performed in vivo microdialysis in NAc and studied voluntary alcohol intake in a two‐bottle choice paradigm and following repeated withdrawal periods.

## 2 Materials and Methods

### 2.1 Animals

Male Wistar Han rats (Inotive, Venray, the Netherlands, *n* = 242) weighing 280–350 g, corresponding to the age of 9–12 weeks, upon arrival, were used in this study. For locomotor activity and in vivo microdialysis experiments, rats were housed under controlled environmental conditions with a 12‐h light/dark cycle (lights on at 7:00 AM and lights off at 7:00 PM). For alcohol consumption studies, animals were housed in a reversed light/dark cycle (lights off at 10 AM and lights on at 10 PM) to align with their active phase during behavioural testing. All animals had ad libitum access to standard rat chow and water. Upon arrival, rats were group‐housed and allowed a 1‐week acclimatization period before the start of any experimental procedures. All experiments were conducted in accordance with protocols approved by the Ethics Committee for Animal Experiments, Gothenburg, Sweden (2401‐19, 3095‐20) and followed the guidelines of the Swedish Legislation on Animal Experimentation (Animal Welfare Act SFS 2018:1192). The methods are reported in accordance with the ARRIVE guidelines.

### 2.2 Drugs

Alcohol (ethanol 95%, Kemetyl AB, Haninge Sweden) was diluted in regular tap water to a final concentration of 6% or 12% (v/v) for alcohol consumption paradigms and diluted in saline (0.9% NaCl w/v) to a concentration of 18% (v/v) for intraperitoneal (i.p.; 2.3 g/kg) administration. Donepezil hydrochloride was dissolved in saline and administered i.p. in doses of 1.5, 3 and 6 mg/kg, based on a brief review of the literature in rats (e.g., [30, 31]). Scopolamine hydrobromide trihydrate and mecamylamine hydrochloride were dissolved in Ringer's solution (140‐mM NaCl, 1.2‐mM CaCl_2_, 3.0‐mM KCl and 1.0‐mM MgCl_2_) to a concentration of 50 μM for local administration into the NAc. Unless otherwise specified, all reagents were purchased from Sigma‐Aldrich.

### 2.3 Locomotor Activity

To evaluate potential dose‐dependent effects of donepezil on spontaneous locomotor activity, rats were tested following systemic administration (i.p.) of different donepezil doses. Rats (*n* = 27) were placed in square test arenas (40 × 40 cm; Med assoc., Fairfax, VT, USA), each housed within a sound‐attenuated, ventilated chamber illuminated with dim light. Locomotor activity was detected using a two‐layered grid of infrared photocell beams. After a 30‐min acclimatization period in the experimental room, baseline locomotion was recorded for 30 min. Donepezil was then administered i.p., and an additional 30‐min recording followed to assess drug‐induced effects. Locomotor data were collected and analysed using Activity Monitor 7 software (Med Assoc., St. Albans, VT, USA).

### 2.4 In Vivo Microdialysis

Implantation of the microdialysis probe was performed as described previously [6]. Briefly, rats were anaesthetised with isoflurane (Baxter, Kista, Sweden) at 4% during induction, placed on a heating pad to prevent hypothermia and mounted onto a stereotactic instrument (David Kopf Instruments, Tujunga, CA, USA). The anaesthesia was continued using isoflurane at 2%–3% throughout the surgery. After exposing the scull, a hole was drilled unilaterally above the target area, the NAc, and two additional holes were drilled for securing anchoring screws. A custom‐made I‐shaped microdialysis probe (molecular weight cut‐off: 20 kDa; active space membrane length: 2 mm) was lowered to coordinates approximating NAc core‐shell borderline region (A/P: +1.85, M/L: ±1.4 relative to bregma, V/D: −7.8 relative to dura mater; Paxinos and Watson 7th compact ed. 2018). Metacam vet (2 mg/mL, 1 mg/kg, subcutaneously; Apoteket AB, Sweden) was given as perioperative analgesia. On the experimental day, the microdialysis probe was connected to a microperfusion pump and perfused with Ringer's solution at a rate of 2 μL/min. A 2‐h equilibration period preceded sample collection. Dialysis samples (40 μL) were collected every 20 min. After obtaining four baseline samples, i.p. injection of donepezil and/or local drug administration was initiated if applicable. Concentrations of DA in the dialysates were analysed using high‐performance liquid chromatography (HPLC) with electrochemical detection, as previously described [32]. Following the experiment, animals were euthanized, and brains were collected and fixed in Accustain Formaline‐free fixative (Sigma‐Aldrich) for 3–7 days. Probe placement was verified through gross anatomical examination of manually sliced sections. Rats with misplaced probe, local bleeding or other brain tissue damage were excluded from the statistical analyses (*n* = 7).

### 2.5 Intermittent Alcohol Consumption

An intermittent two‐bottle choice paradigm was used to assess voluntary alcohol consumption. Rats were provided access to alcohol three times per week (Monday, Wednesday and Friday) for 24‐h sessions, beginning at the onset of the dark cycle (10:00 AM) to align with natural activity rhythms. Initially, animals received 6% (v/v) alcohol for 2 weeks, followed by 12% (v/v) alcohol for an additional 3 weeks. After this screening period, rats demonstrating consistent alcohol intake of at least 1.5 g/kg/24 h (a priori inclusion criterion; *n* = 6 did not meet this criterion) were randomized into two groups: donepezil (3 mg/kg; *n* = 14) or vehicle (saline; *n* = 14). The groups were stratified to ensure similar pretreatment alcohol intake levels. Treatment was administered i.p. 30 min prior to each alcohol session, continuing for 12 consecutive sessions (4 weeks) using the 12% alcohol solution. Fluid intake was measured daily, and body weight was monitored weekly. Pre‐set exclusion criterion was leaking bottles; no animals were excluded after treatment started.

### 2.6 Alcohol Deprivation Effect (ADE)

To assess the effect of donepezil on relapse‐like drinking, a separate drinking paradigm with a different set of animals was performed to study the ADE. The animals were presented with an intermittent two‐bottle choice alcohol consumption paradigm with three 24‐h sessions per week and three rounds of deprivation periods. The animals were habituated to 6% (v/v) alcohol solution over three sessions (1 week), after which the concentration was increased to 12% (v/v). After 3 weeks, rats demonstrating consistent alcohol intake of at least 1.5 g/kg/24 h (a priori inclusion criterion; no rats were excluded) were selected for further studies. During the following 2 weeks, the animals were deprived of alcohol, after which alcohol was reintroduced with intermittent access to alcohol for three 24‐h sessions a week for 2 weeks. The deprivation cycles were repeated, and donepezil treatment (3 mg/kg) (*n* = 15) or vehicle (*n* = 15) was given following the third alcohol deprivation period to ensure that a robust ADE was established. Pre‐set exclusion criterion was leaking bottles; *n* = 1 was excluded from the donepezil group due to a leaking bottle.

### 2.7 Statistics

All statistical analyses were performed using GraphPad Prism Software version 10 (GraphPad Software Inc. San Diego, CA, USA). Two‐way repeated‐measure analysis of variance (ANOVA) was used to analyse alcohol and water intake over time. One‐way ANOVA was conducted on ambulatory and vertical counts for measurement of locomotor activity and on area under the curve (AUC) data from microdialysis experiments. For these analyses, Tukey's post hoc test was used. Unpaired *t*‐tests were used to compare average alcohol and water intake (g/kg/24 h) between groups during the treatment period and to compare AUC data from a subset of microdialysis experiments. Mixed‐effects modeling was employed for ADE data analysis, and uncorrected Fisher's LSD test was used for post hoc analyses. Data are presented as mean ± standard error of the mean (SEM), and a *p*‐value < 0.05 was considered statistically significant.

## 3 Results

### 3.1 Locomotor Activity Is Not Affected by Donepezil

**FIGURE 1:**
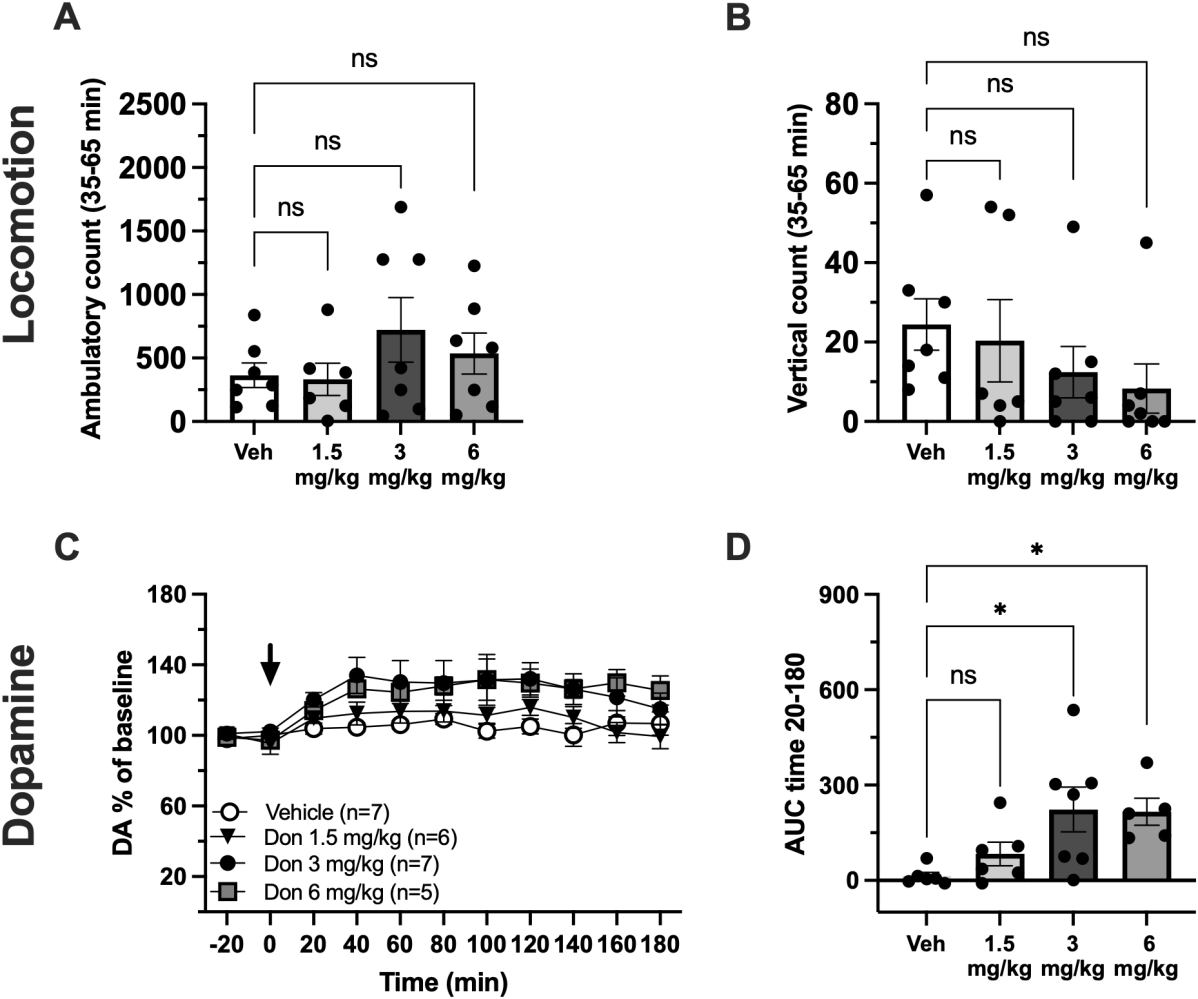
Donepezil elevates extracellular dopamine within the nucleus accumbens. (A) Cumulative ambulatory counts over 30 min following donepezil treatment (timepoints 35–65 min). (B) Cumulative vertical counts over 30 min following donepezil treatment (timepoints 35–65 min). No significant differences in locomotor activity were observed for any dose of donepezil compared to vehicle (A, B). (C) Time course graph of dopamine (DA) % of baseline. (D) Dopamine data presented as area under the curve (AUC) for timepoints 20–180 min. A significant increase in DA was seen following treatment with donepezil 3 and 6 mg/kg. Arrows indicate administration of donepezil or vehicle (i.p.). ns = nonsignificant, * *p* < 0.05.

Locomotor activity was assessed following i.p. administration of vehicle or donepezil at doses of 1.5, 3 and 6 mg/kg. No significant differences in ambulatory counts were observed between treatment groups (one‐way ANOVA, counts over timepoints 35–65 min; treatment effect *F*_(3, 23)_ = 1.058, *p* = 0.386) (Figure 1A). Similarly, no significant difference in rearing activity was detected (one‐way ANOVA, counts over timepoints 35–65 min; treatment effect *F*_(3, 23)_ = 1.022, *p* = 0.401) (Figure 1B). Although no statistically significant change in locomotor behaviour was detected using these measures, animals treated with the highest dose (6 mg/kg) exhibited clear signs of peripheral cholinergic stimulation, including diarrhoea, salivation and fasciculations, well‐documented side effects of excessive cholinergic activation [33]. These effects were not observed at the lower doses (1.5 or 3 mg/kg), suggesting that the 6‐mg/kg dose may produce systemic side effects.

### 3.2 Systemic Administration of Donepezil Increases Extracellular Levels of Dopamine in the NAc

Systemic administration of donepezil at three different doses (1.5, 3 and 6 mg/kg, i.p.) was evaluated for its effect on extracellular DA in the NAc using in vivo microdialysis. Donepezil was administered at timepoint 0, and DA levels were monitored for 180 min. Analysis of the AUC revealed a significant effect of treatment (one‐way ANOVA for AUC over timepoints 20–180; *F*_(3, 20)_ = 4.405, *p* = 0.016). Post hoc comparisons showed that 3‐ and 6‐mg/kg donepezil significantly increase extracellular DA levels compared to vehicle (Tukey's test, *p* = 0.014 and *p* = 0.030, respectively) (Figure 1C,D). The lowest dose, 1.5 mg/kg, produced no significant change in DA levels compared to vehicle (Tukey's test, *p* = 0.630) (Figure 1C,D). The 3‐mg/kg dose significantly elevated DA levels in the NAc without affecting locomotor activity (Figure 1) and thus offered an optimal balance between efficacy and tolerability and was therefore used for subsequent experiments.

### 3.3 Donepezil‐Induced Dopamine Increase Is Blocked Using a Muscarinic Antagonist

**FIGURE 2:**
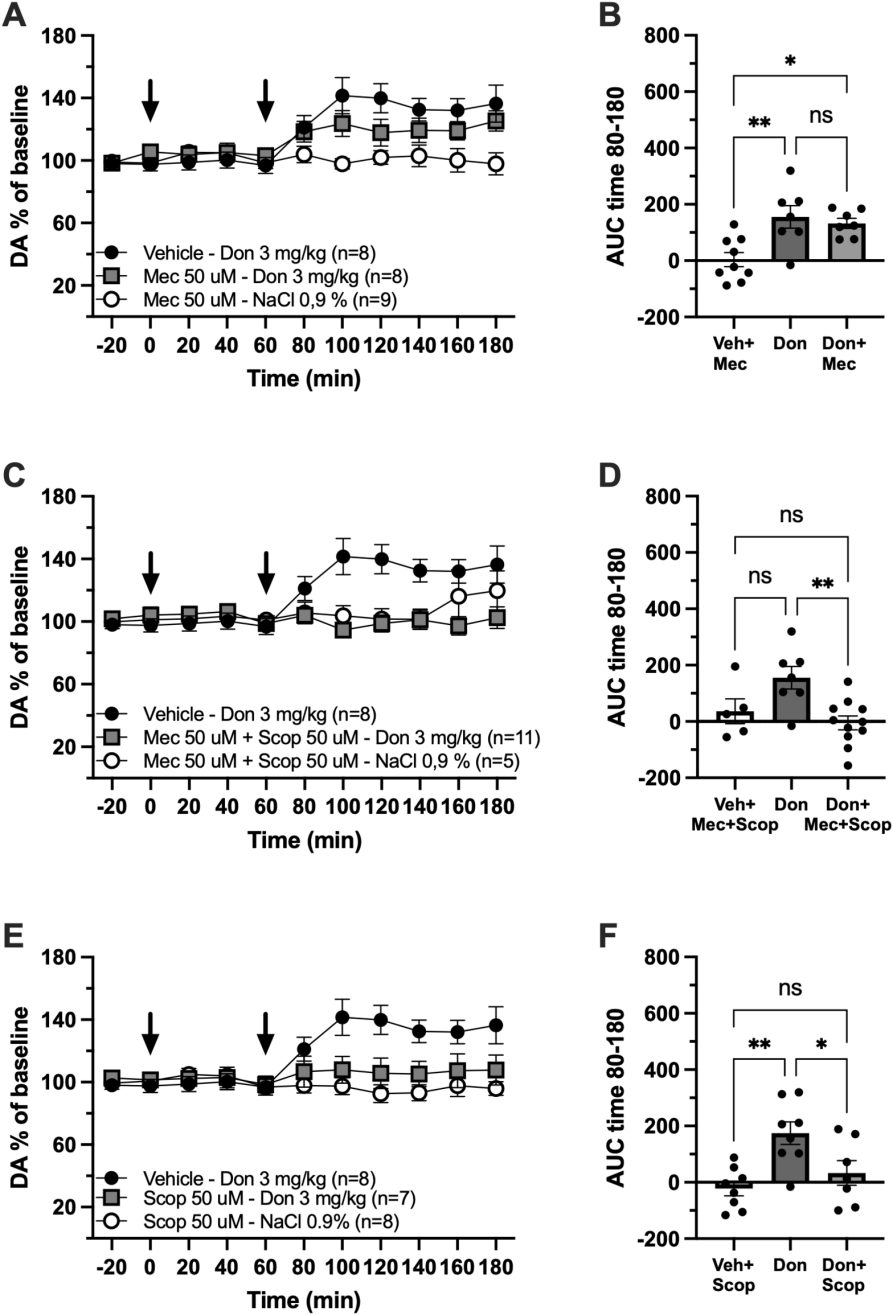
Donepezil‐induced dopamine release is blocked by antagonism of accumbal muscarinic receptors. (A) Time course graph presented as dopamine (DA) % of baseline, arrows indicate start of drug perfusion at timepoint 0 and i.p. injection of donepezil (Don) or vehicle at timepoint 60. (B) Data presented as area under the curve (AUC) over time 80–180 min. Donepezil‐induced DA increase was partly but not significantly blocked by mecamylamine (Mec). (C) Time course graph presented as DA % of baseline, arrows indicate start of drug perfusion at timepoint 0 and i.p. injection of donepezil or vehicle at timepoint 60. (D) Data presented as area under the curve (AUC) over time 80–180 min. Donepezil‐induced DA increase was fully blocked by antagonism of both muscarinic and nicotinic receptors. (E) Time course graph presented as DA % of baseline, arrows indicate start of drug perfusion at timepoint 0 and i.p. injection of donepezil or vehicle at timepoint 60. (F) Data presented as area under the curve (AUC) over time 80–180 min. Donepezil‐induced DA increase was fully blocked by scopolamine (Scop) pretreatment. All data presented as mean ± standard error of mean, *n* = number of animals, ns = nonsignificant, * *p* < 0.05, ** *p* < 0.01.

To investigate whether the donepezil‐induced increase in extracellular DA within the NAc is mediated by local cholinergic receptors, in vivo microdialysis combined with local pharmacological manipulation (reversed dialysis) was employed. Local perfusion of the nAChR antagonist mecamylamine (50 μM) into the NAc prior to systemic donepezil administration (3 mg/kg, i.p.) did not block the donepezil‐induced DA increase (one‐way ANOVA for AUC over timepoints 80–180; *F*_(2, 20)_ = 8.64, *p* = 0.002; Tukey's post hoc test: donepezil vs. donepezil + mecamylamine, *p* = 0.857) (Figure 2A,B). Next, simultaneous local perfusion of mecamylamine (50 μM) and the mAChR antagonist scopolamine (50 μM) was performed prior to systemic donepezil administration (3 mg/kg, i.p.). This combination produced a complete blockade of the donepezil‐induced DA release (one‐way ANOVA for AUC over timepoints 80–180; *F*_(2, 20)_ = 6.459, *p* = 0.007; Tukey's post hoc test: donepezil vs. donepezil + scopolamine + mecamylamine, *p* = 0.005) (Figure 2C,D). Finally, local perfusion of scopolamine (50 μM) alone, prior to systemic administration of donepezil (3 mg/kg, i.p.), also resulted in a full blockade of the DA response (one‐way ANOVA for AUC over timepoints 80–180; *F*_(2, 20)_ = 7.412, *p* = 0.003; Tukey's post hoc test: donepezil vs. donepezil + scopolamine, *p* = 0.036) (Figure 2E,F).

### 3.4 Alcohol‐Induced Dopamine Release Is Blunted in Rats Pretreated With Donepezil

**FIGURE 3:**
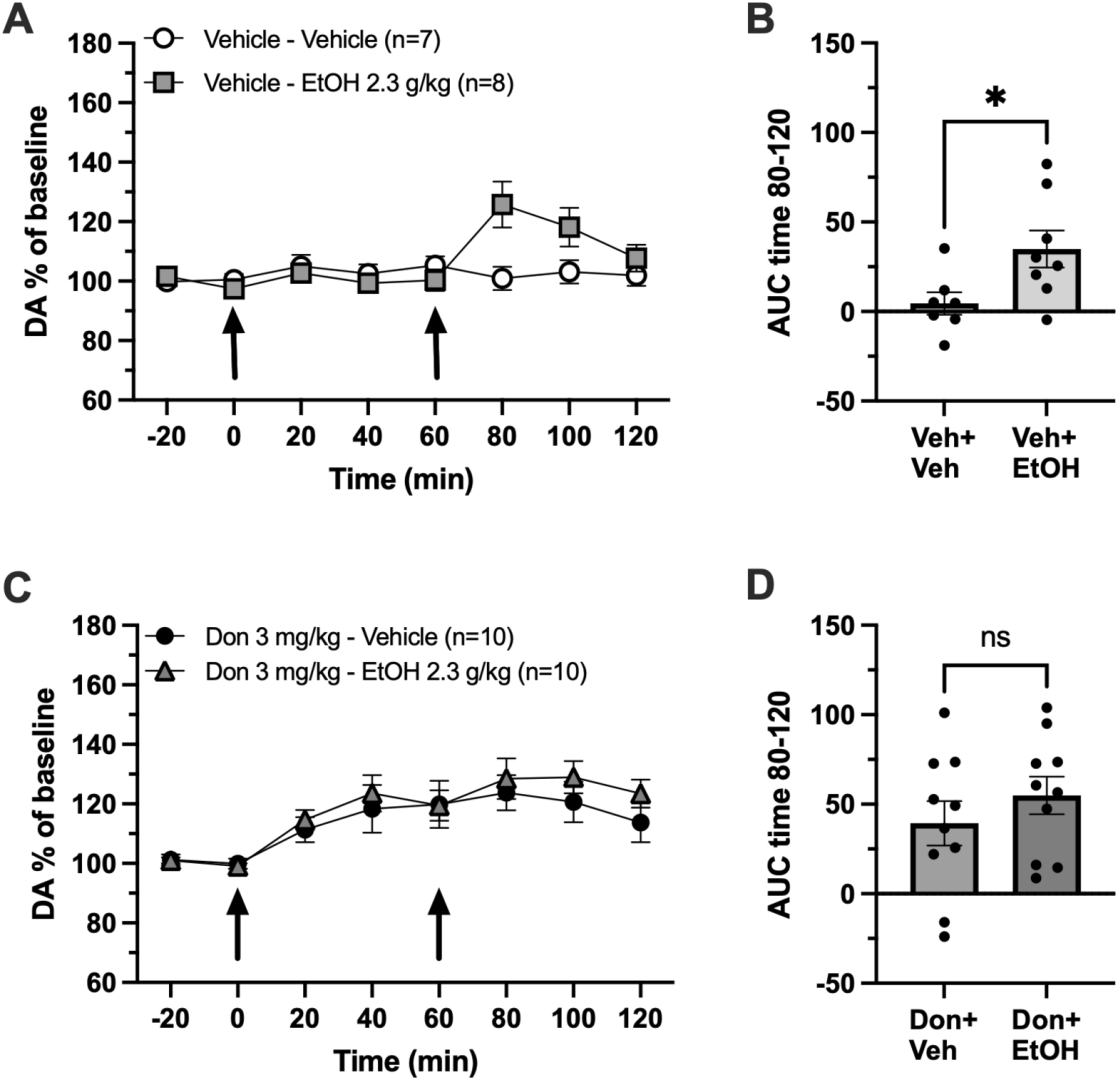
Donepezil occludes alcohol‐induced dopamine increase. (A) Time course graph presented as dopamine (DA) % of baseline, arrows indicate i.p. injection of vehicle at timepoint 0 and alcohol (EtOH) 2.3 g/kg or vehicle at timepoint 60. (B) Data presented as area under the curve (AUC) over time 80–120 min. (C) Time course graph presented as dopamine (DA) % of baseline, arrows indicate i.p. injection of donepezil (Don) 3 mg/kg at timepoint 0 and alcohol (EtOH) 2.3 g/kg or vehicle at timepoint 60. (D) Data presented as area under the curve (AUC) over time 80–120 min. All data presented as mean ± standard error of mean, *n* = number of animals, ns = nonsignificant, * *p* < 0.05.

Donepezil increased basal dopamine levels in the NAc (Figure 1C,D). To evaluate potential additive or interactive effects of alcohol and donepezil on extracellular DA levels, DA responses were compared between donepezil treatment alone and combined treatment with donepezil and alcohol. A significant increase in extracellular DA levels in the NAc was seen following alcohol administration (unpaired *t*‐test for AUC over timepoints 80–120, *t*_(13)_ = 2.419, *p* = 0.031) (Figure 3A,B). No significant difference was observed between donepezil treatment and donepezil together with alcohol (unpaired *t*‐test for AUC over timepoints 80–120, *t*_(18)_ = 0.953, *p* = 0.353) (Figure 3C,D).

### 3.5 Donepezil Treatment Does Not Affect Intermittent Alcohol Intake or Water Consumption

**FIGURE 4:**
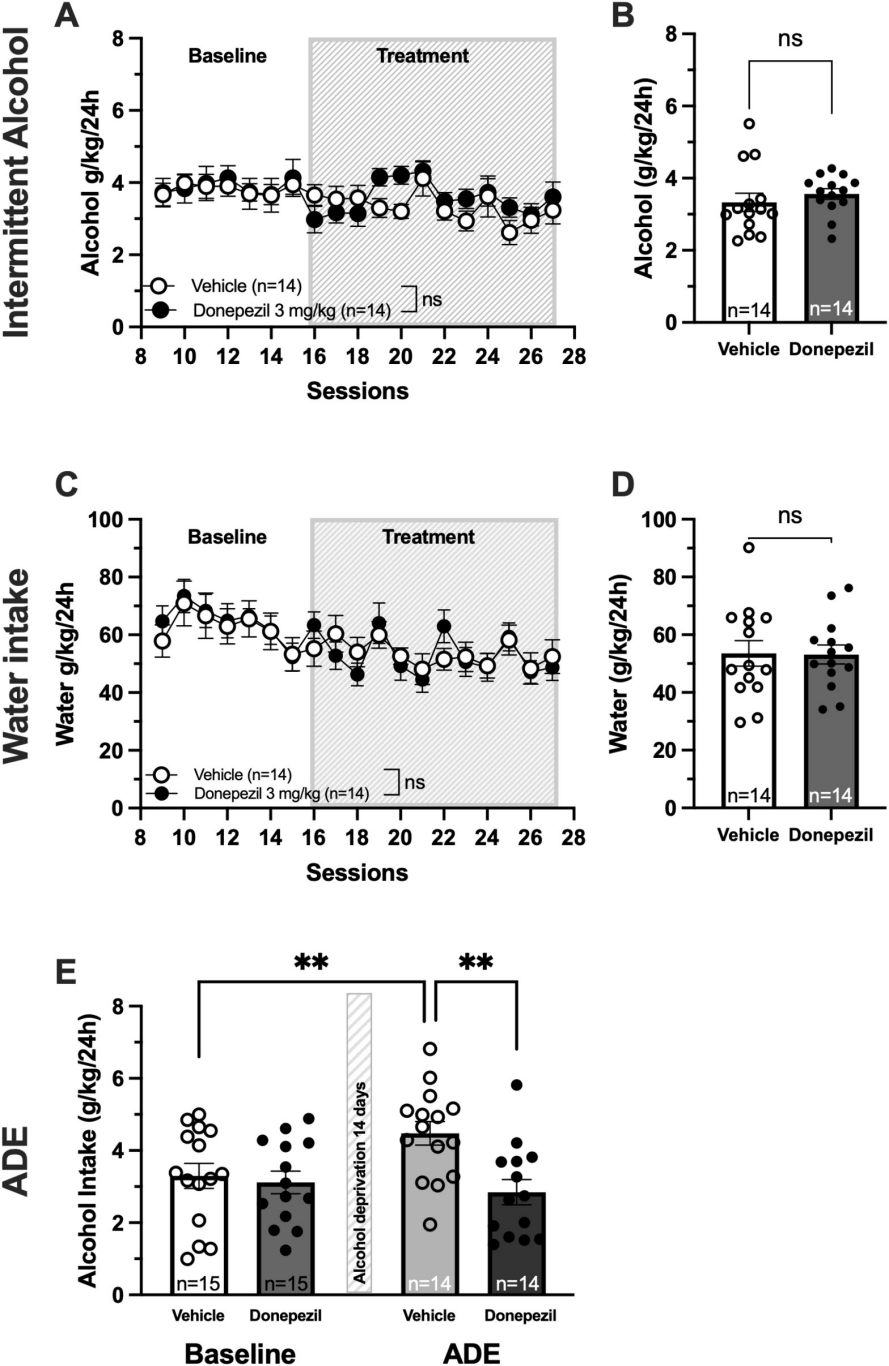
Donepezil treatment (3 mg/kg) abolishes the alcohol deprivation effect. (A) Time course graph of alcohol intake (g/kg/24 h). (B) Mean alcohol intake (g/kg/24 h) over the treatment period (sessions 16–27). No significant difference in alcohol intake was observed between vehicle‐ and donepezil‐treated rats (A, B). (C) Time course graph of water intake (g/kg/24 h). (D) Mean water intake (g/kg/24 h) over the treatment period (sessions 16–27) did not differ between treatment groups. (E) An alcohol deprivation effect (ADE) was observed in vehicle‐treated animals but not in donepezil‐treated rats, data presented as baseline (mean alcohol intake over the last three sessions before alcohol deprivation) vs. first drinking session following alcohol deprivation. All data presented as mean ± standard error of mean, *n* = number of animals, ns = nonsignificant, ** *p* < 0.01.

To assess the effects of donepezil on voluntary ethanol consumption, rats were subjected to an intermittent two‐bottle choice alcohol drinking paradigm. Donepezil treatment (3 mg/kg, i.p., 30 min prior to alcohol access) did not significantly alter 24‐h alcohol intake compared to vehicle‐treated controls (two‐way ANOVA of sessions 16–27; group effect *F*_(2, 40)_ = 0.900, *p* = 0.4169; *t*‐test of mean alcohol intake (g/kg/24 h) over sessions 16–27; *t*_(26)_ = 0.793, *p* = 0.435) (Figure 4A,B). No effect of donepezil was observed on water intake (two‐way ANOVA of sessions 16–27; group effect *F*_(2, 40)_ = 0.079, *p* = 0.924; *t*‐test of mean water intake (g/kg/24 h) over sessions 16–27; *t*_(26)_ = 0.075, *p* = 0.941) (Figure 4C,D).

### 3.6 Donepezil Treatment Abolishes the ADE

To evaluate the effect of donepezil on relapse‐like drinking behaviour, a separate ADE paradigm was employed. Following three repeated 2‐week deprivation periods, vehicle‐treated rats displayed a significant increase in alcohol intake compared to baseline, consistent with the ADE (mixed‐effect analysis; treatment effect *F*_(1, 27)_ = 5.486, *p* = 0.027, treatment × time effect *F*_(1, 27)_ = 7.099, *p* = 0.013; uncorrected Fisher's LSD test: vehicle baseline vs. vehicle ADE, *p* = 0.004). In contrast, donepezil‐treated rats (3 mg/kg) exhibited a blunted ADE response (uncorrected Fisher's LSD test: donepezil baseline vs. donepezil ADE, *p* = 0.495; ADE vehicle vs. ADE donepezil [3 mg/kg] *p* = 0.001) (Figure 4E). No significant difference in baseline alcohol intake was observed between treatment groups (uncorrected Fisher's LSD test: baseline vehicle vs. baseline donepezil, *p* = 0.706).

## 4 Discussion

Cholinergic signaling plays a key role in modulating DA transmission within the NAc, a process closely linked with the rewarding and reinforcing effects of addictive substances, including alcohol. In this study, we demonstrate that systemic administration of the AChEI donepezil increases extracellular DA levels in the NAc and abolishes the ADE in male rats. Given that the ADE model is considered predictive of relapse‐like drinking in humans with AUD [34], our findings suggest that donepezil may modulate neurobiological mechanisms underlying relapse behaviour.

The observation that donepezil at 3 mg/kg significantly elevated extracellular DA in the NAc aligns with previous findings showing that AChEI enhances DA release both in vitro [35] and in vivo [6]. The most likely mechanism involves increased synaptic ACh levels due to acetylcholinesterase inhibition, leading to enhanced cholinergic tone. Although we did not directly confirm this mechanism, it is consistent with the established role of CINs in the NAc in stimulating local DA release via nAChRs [14, 15, 16]. However, local antagonism of nAChRs with mecamylamine did not significantly attenuate the donepezil‐induced DA increase. This finding contrasts with studies emphasizing a key role for nAChRs in ACh‐induced DA release. Methodological differences may explain this discrepancy. The 20‐min sampling intervals used in in vivo microdialysis mainly measure tonic DA release and thus cannot capture the rapid, phasic DA release typically governed by nAChR signaling. Moreover, most previous studies are ex vivo and have used stimulating techniques, whereas our study was a pharmacology‐based in vivo study. Additionally, prolonged exposure to ACh can desensitize nAChRs [35, 36], potentially limiting mecamylamine's effect under these conditions. It should be noted that only a single dose of mecamylamine was used, and a higher dose might produce a different outcome. In contrast, pretreatment with the muscarinic antagonist scopolamine, alone or in combination with mecamylamine, fully blocked the donepezil‐induced DA increase. This finding is consistent with earlier work showing that physostigmine‐induced DA elevation was abolished by scopolamine [6] and supports a key role for mAChRs in this effect. Activation of mAChRs has been shown to enhance DA release both in vitro [37, 38] and in vivo [39]. Among the mAChR subtypes (M_1_‐M_5_), the M_5_ receptor is uniquely expressed on midbrain DA neurons [40] and has been implicated in facilitating DA release upon activation [39, 41]. While our results suggest involvement of mAChRs, further studies are needed to confirm the specific subtype mediating this effect.

Our group has previously shown that local administration of scopolamine and/or mecamylamine does not affect basal NAc DA levels [6, 8], suggesting that cholinergic signaling is not required to maintain basal DA tone. Consistent with these earlier findings, the present study also detected no changes in basal DA levels following treatment with scopolamine, mecamylamine or their combination (Figure 2A–C).

Reduced DA neurotransmission has been associated with heightened craving and alcohol intake in both clinical populations [42] and preclinical models [19, 43]. Pharmacological strategies that modestly elevate tonic DA in the NAc may reduce the motivational salience of alcohol cues and thus hold therapeutic potential for AUD. Although donepezil did not affect voluntary alcohol consumption during intermittent access, it robustly abolished the ADE, indicating that it may prevent relapse following abstinence rather than affect ongoing consumption. Notably, alcohol administration following donepezil treatment did not further increase extracellular DA levels, potentially reducing the reinforcing effects that drive excessive intake. The neurochemical profile resembles that of approved AUD medications; acamprosate, for example, increases NAc DA while preventing additional alcohol‐induced DA increase [44] and also blocks the ADE in rats [45]; naltrexone, a relapse‐preventing treatment [46] that reduces alcohol craving in humans [47], similarly inhibits alcohol‐induced DA increases in the NAc [48]. These parallels suggest that donepezil may share beneficial pharmacodynamic properties with existing AUD treatments.

The therapeutic relevance of cholinergic modulation in AUD is further supported by studies on other agents. The partial nAChR agonist varenicline increases DA levels in the NAc [26] and reduces alcohol intake in both preclinical [25] and clinical settings [27, 28]. Combined varenicline and bupropion treatment produces additive DA release and synergistically reduces the ADE in rats [26]. Similarly, galantamine, an AChEI with a modulatory effect on nAChRs, has been shown to decrease alcohol consumption in individuals with AUD [29]. Together, these findings underscore that controlled enhancement of DA signaling via cholinergic mechanisms can reduce alcohol intake and relapse risk.

Donepezil treatment in individuals with AUD may offer benefits that extend beyond reducing alcohol intake. Evidence suggests that donepezil can protect against alcohol‐induced neurotoxicity and may therefore exert a neuroprotective effect against alcohol‐induced neuronal damage [49, 50, 51]. In addition, donepezil may help alleviate alcohol‐induced cognitive impairments [52] and improve alcohol‐related delirium [53] in clinical settings. Because executive cognitive function and memory play roles in motivation and the regulation of drinking behaviour, these cognitive benefits may further support its therapeutic value in AUD.

This study has some limitations. Sex differences in AUD pathophysiology and progression have been documented, underscoring the need to include both males and females in preclinical and clinical alcohol studies. The exclusive use of males in the present study is therefore a limitation, and replication in females will be necessary to determine whether these effects generalize across sexes. A limitation of the dose–response experiments is that alcohol‐naïve animals were used in the locomotor activity tests, whereas rats with prior alcohol exposure may be more sensitive to the side effects of donepezil. Another limitation is that the present work focused solely on the role of AChRs within the NAc in mediating donepezil‐induced DA release. Future studies should also examine AChRs in other key regions of the mesolimbic pathway, such as the VTA, to provide a more comprehensive understanding of cholinergic modulation of DA signaling.

In conclusion, these findings indicate that cholinergic modulation, particularly through muscarinic mechanisms in the NAc, plays a critical role in regulating DA levels and relapse‐like alcohol consumption. Systemic administration of donepezil increased extracellular DA in the NAc and abolished ADE in rats, supporting its potential as a pharmacological strategy for relapse prevention in AUD. Future studies should identify the receptor subtypes mediating these effects and evaluate the translational potential of donepezil and related compounds in clinical populations.

## Author Contributions

**Anna Loftén:** conceptualization, investigation, formal analysis, writing – original draft, funding acquisition. **Klara Danielsson:** investigation, formal analysis. **Louise Adermark:** conceptualization, writing – review and editing, funding acquisition. **Bo Söderpalm:** conceptualization, writing – review and editing, supervision, funding acquisition. **Mia Ericson:** conceptualization, writing – review and editing, supervision, funding acquisition.

## Funding

This work has been financially supported by Göteborgs Läkaresällskap (GLS‐1000918 and GLS‐1022159), Wilhelm och Martina Lundgrens Vetenskapsfond (2024‐SA‐4659), Hjärnfonden, the Swedish Medical Research Council (Dnr: 2020‐00559, 2020‐01346 and 2020‐02105) and governmental support from LUA/ALF.

## Ethics Statement

All experiments were conducted in accordance with protocols approved by the Ethics Committee for Animal Experiments, Gothenburg, Sweden (2401‐19, 3095‐20).

## Conflicts of Interest

B.S. and M.E. own stock in Sobrera Pharma AB, a company developing new pharmaceutical treatments for AUD. B.S. has received honoraria for lectures from Lundbeck, Takeda and Evolan. The authors have no other conflicts of interest in relation to the subject of this study.

## Data Availability

The dataset generated during the current study is available from the corresponding author on reasonable request.
